# The blue light signaling inhibitor 3-bromo-7-nitroindazole affects gene translation at the initial reception of blue light in young *Arabidopsis* seedlings

**DOI:** 10.5511/plantbiotechnology.24.0323a

**Published:** 2024-06-25

**Authors:** Yukio Kurihara, Chika Akagi, Yuko Makita, Masaharu Kawauchi, Emiko Okubo-Kurihara, Tomohiko Tsuge, Takashi Aoyama, Minami Matsui

**Affiliations:** 1 Synthetic Genomics Research Group, RIKEN Center for Sustainable Resource Science; 2 Department of Life Sciences, Graduate School of Arts and Sciences, The University of Tokyo; 3 Institute for Chemical Research, Kyoto University; 4 Faculty of Engineering, Maebashi Institute of Technology; 5 Rikkyo University, College of Science; 6 Graduate School of Nanobioscience, Department of Life and Environmental System Science, Yokohama City University

**Keywords:** initial reception of blue light, Ribo-seq, 3B7N, translatome

## Abstract

Initial light reception after germination is a dramatic life event when a seedling starts proper morphogenesis. Blue light contains a range of light wavelengths that plants can perceive. A previous report suggested that the chemical compound 3-bromo-7-nitroindazole (3B7N) inhibits blue light-mediated suppression of hypocotyl elongation by physically interacting with the blue light receptor Cryptochrome 1 (CRY1). We previously examined changes of genome-wide gene expression in *Arabidopsis* seedlings germinated in the dark and then exposed to blue light by RNA-seq and Ribo-seq analyses. The expression of ribosome-related genes was translationally upregulated in response to the initial blue light exposure, depending on signals from both the nucleus and chloroplasts. Here, we re-analyzed our previous data and examined the effect of 3B7N treatment on changes in gene expression upon blue light exposure. The results showed that 3B7N negatively affected translation of ribosome-related genes and, interestingly, the effects were similar to not only those in *cry1cry2* mutants but also plants under suppression of photosynthesis. We propose an apparent crosstalk between chloroplast function and blue light signaling.

Following germination in the soil (in the dark), plants initiate a process called photomorphogenesis, which includes the arrest of hypocotyl elongation and development of cotyledons, when they first receive light on the ground. Dramatic changes in gene expression occur during this first photoreception (Kurihara et al. 2018, 2020a, 2020b, 2022), but the regulatory mechanism is still largely unknown.

The *Arabidopsis* genome encodes multiple genes for photoreceptors that can accept various wavelengths of light, such as red, far-red, and blue light. Among them, two cryptochromes, CRY1 and CRY2, specifically accept blue light. Loss-of-function mutants of *CRY1* and *CRY2* exhibit hypocotyl elongation under blue light compared with wild-type (WT) plants due to inhibition of blue light signal transmission (Ong et al. 2017). ELONGATED HYPOCOTYL5 (HY5) serves as a hub regulator in the light signaling pathway (Gangappa and Botto 2016; Xiao et al. 2022). Under blue light, *hy5* mutants also exhibit a long hypocotyl phenotype like *cry1cry2* mutants (Ong et al. 2017).

To understand how blue light signaling and chloroplast function are involved in expression of nuclear genes when etiolated seedlings are exposed to blue light, we performed comparative RNA-seq and ribosome profiling (Ribo-seq) analyses in the WT, *cry1cry2* and *hy5* mutants and plants treated with inhibitors of photosynthesis (3-(3,4-dichlorophenyl)-1,1-dimethylurea: DCMU) (Petrillo et al. 2014) and chloroplast development (norflurazon) (Park et al. 2017). This work revealed that expression of ribosome-related genes is translationally upregulated in response to blue light exposure, dependent on signals from both the nucleus and chloroplasts (Akagi et al. 2023).

In this paper, we first re-analyzed and re-evaluated our previous RNA-seq and Ribo-seq data (Akagi et al. 2023) (Supplementary Table S1). Ribo-seq analysis, which captures and sequences ribosome footprints, reveals genome-wide translational states (Fujita et al. 2019; Ingolia et al. 2009). In our previous study, seeds were sown on filter paper (Toyo Roshi Kaisha) placed on agar medium, allowed to grow in the dark for 3 days, and then exposed to blue light (6.32 µmol m^−2^ s^−1^) for 3 h. For chemical treatment of WT plants, agar medium was pre-filled with DCMU or norflurazon at a final concentration of 15 µM. The seedlings were harvested before and after blue light exposure for RNA-seq and Ribo-seq analyses. In the previous analysis, expression levels were calculated for open reading frames (ORFs) predicted by a computational program, RiboTaper (Calviello et al. 2016). However, in this re-analysis, expression levels for each gene were obtained based on the Araport11 gene model (TAIR), which has been broadly used in some previous studies to avoid the uncertainty associated with ORF prediction and uneven numbers of detected genes among genotypes or treatments by RiboTaper. Besides, use of Araport11 has the advantage of quantifying expression level against a defined gene model across all datasets. To avoid duplicate counts of the same gene, expression data for mRNA-generating genes including protein-coding and transposable element genes with a variant number of 1 for each gene were used for subsequent analysis. There were few variations between replicates of each genotype or chemical treatment (Supplementary Figure S1), and between the expression changes of RiboTaper-predicted and Araport11-derived ORFs upon blue light exposure (Supplementary Figure S2), supporting the reliability of this re-analysis.

Translation efficiency (TE) refers to the number of Ribo-seq reads (ribosome footprints) divided by the number of RNA-seq reads for each gene; that is, the amount of translation per mRNA (Fujita et al. 2019; Ingolia et al. 2009). The re-analysis identified 656 genes with increased TE (TE-up-regulated genes) and 928 genes with increased mRNA accumulation (mRNA-up-regulated genes) upon blue light exposure in the WT (Table 1 and Supplementary Tables S2, S3 and S4). For gene ontology (GO) enrichment analysis, a set of at least hundreds of genes is required; thresholds of *q*-value <0.05 and *p*-value <0.01 were used to determine TE-up-regulated and mRNA-up-regulated genes, respectively (Table 1 and Supplementary Table S1). GO enrichment analysis showed that ribosome-related and chloroplast-related GO terms were enriched for the TE-upregulated genes (Figure 1A), consistent with our previous report (Akagi et al. 2023). On the other hand, chloroplast-related and light response-related GO terms were enriched for the mRNA-upregulated genes (Figure 1B). The subcellular localization of the proteins encoded by these genes was examined. Both TE- and mRNA-upregulated genes encoded more chloroplast-localized proteins than the proportion of all genes (Figure 1C). Importantly, however, the overlap between TE- and mRNA-upregulated genes was quite small (Figure 1D). These results suggest that chloroplast-related genes are likely regulated either transcriptionally or translationally, but not both, at initial photoreception.

**Table 1:** Table 1. Numbers of mRNA- or TE-up-regulated genes upon blue light exposure.

	*p*- or *q*-value	WT	*hy5*	*cry1cry2*	DCMU	Norf.	3B7N
mRNA-up-regulated genes	*p*<0.01*	928	628	701	1154	933	627
TE-up-regulated genes	*q*<0.05**	656	194	213	229	471	77

Norf., norflurazon, *two-tailed Student’s *t*-test, **p-values were adjusted using Benjamini–Hochberg method.

**Figure 1:**
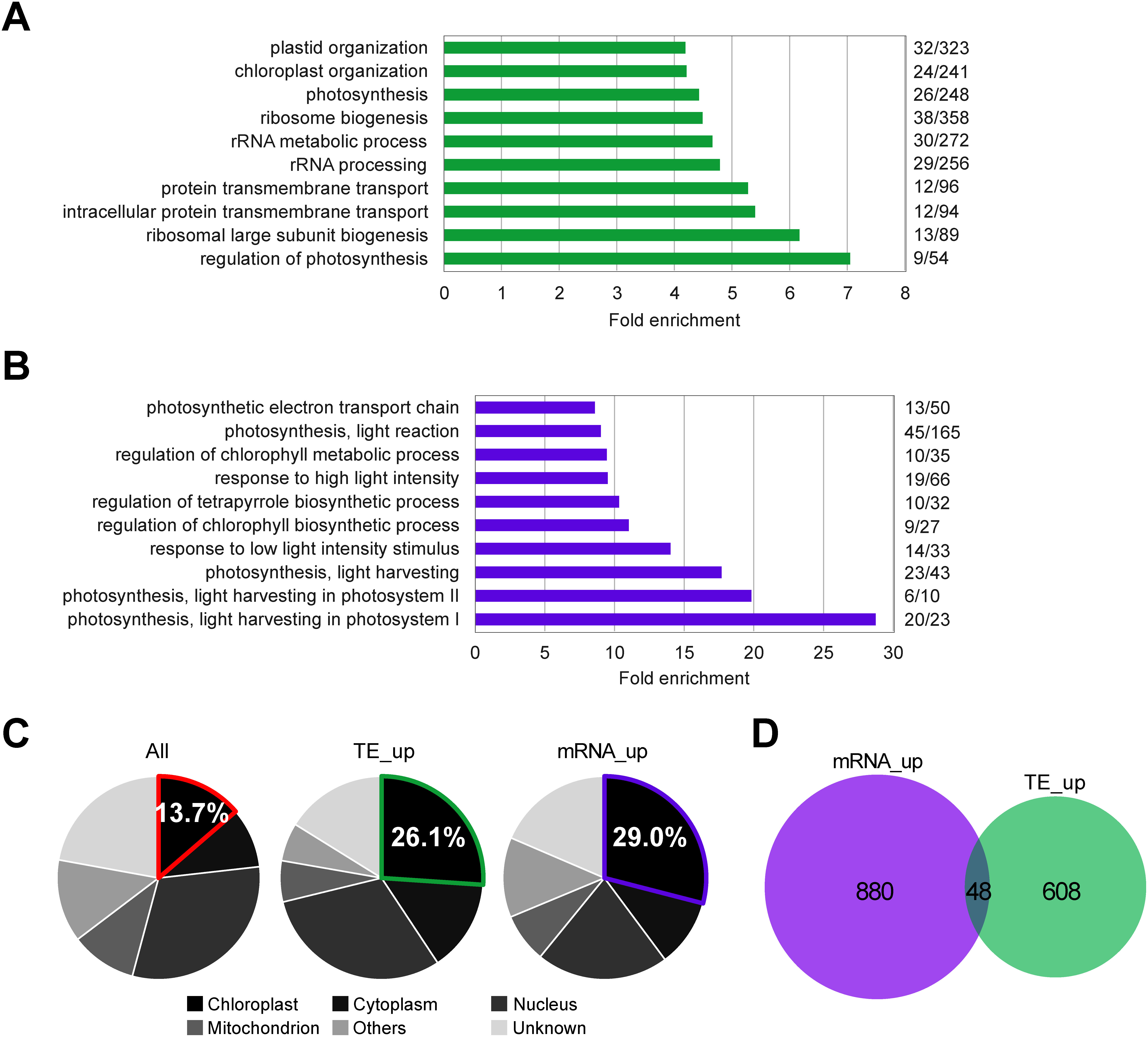
Figure 1. TE- and mRNA-up-regulated genes upon 3 h blue light exposure from darkness. (A) Gene ontology (GO) analysis of TE-up-regulated genes in the WT. (B) GO analysis of mRNA-upregulated genes in the WT. GO analysis was performed using Fisher’s exact test with Bonferroni correction (*p*-value <0.05). Numbers of enriched genes relative to total numbers of genes in each GO term are shown on the right. (C) Localization prediction of products of the TE- and mRNA-up-regulated genes and all genes. (D) Overlap between TE- and mRNA-up-regulated genes.

Our previous chemical screening identified a chemical compound, 3-bromo-7-nitroindazole (3B7N), that inhibits blue light-mediated suppression of hypocotyl elongation by physically interacting with a blue light receptor, CRY1 (Ong et al. 2017). Subsequently, Orth et al. (2017) revealed that binding of ATP to CRY1 promotes the photoreduction of flavin, but 3B7N competes with ATP for binding, thus inhibiting the photoreduction. These findings suggest that 3B7N functions as an inhibitor of blue light signaling. Here, RNA-seq and Ribo-seq analyses were performed on 3B7N (Fujifilm)-treated WT seedlings under the same dark-to-blue light conditions as above, and the results were evaluated with the previous reanalyzed data (WT, *cry1cry2*, *hy5*, DCMU and norflurazon). For this purpose, lysate preparation, cycloheximide treatment, total RNA extraction and library construction for two replicates of Ribo-seq and RNA-seq were done as described previously (Akagi et al. 2023; Kurihara et al. 2018). The libraries were sequenced on a HiSeq X platform (Illumina). The sequenced data were deposited in the DDBJ/EMBL/GenBank BioProject under accession number DRA014541 together with the previous data. The raw reads of RNA-seq and Ribo-seq were mapped onto the *Arabidopsis* TAIR10 genome using STAR (Dobin et al. 2013) and Tophat version 2.1.1 (Trapnell et al. 2009), respectively, with the default random mapping after removal of reads derived from rRNA/tRNA. Normalized expression values for genes were calculated using DESeq (Anders and Huber 2010). The distribution peak of ribosomal footprints for each analysis was 28 nt and 3-nt periodicity along ORFs, a characteristic of ribosomal footprints, was detected (Supplementary Figure S3).

The numbers of TE- or mRNA-upregulated genes in the *hy5* and *cry1cry2* mutants and plants treated with DCMU, norflurazon and 3B7N are summarized in Table 1 (Supplementary Tables S3 and S4). 3B7N treatment more dramatically reduced the number of TE-upregulated genes upon blue light exposure than the other mutations and treatments. To further determine the effects of the inhibitors and mutations on gene expression, the 656 TE-upregulated genes in the WT were examined. The increase in their TE was substantially suppressed by the *cry1cry2* and *hy5* mutations and inhibitor treatments including 3B7N treatment (Figure 2A and Supplementary Figure S4A), while there was a relatively small difference in the change in mRNA accumulation except under norflurazon treatment (Figure 2B and Supplementary Figure S4B). A principal component analysis for the changes in TE of the 656 genes showed that, interestingly, the change under 3B7N treatment was similar not only to that in *cry1cry2* and *hy5* mutants, but also to that under DCMU treatment (Figure 2C). Of the 656 genes, 446 genes were not significantly upregulated in *cry1cry2* plants or plants treated with DCMU or 3B7N (Figure 2D and Supplementary Table S5). Comparing the GO analysis for these 446 genes with Figure 1A, we found that the 446 genes contained most of the ribosome-related genes for which TE was increased in the WT (Figure 2E). These results suggest that inhibition of blue light signaling and chloroplast function negatively affects the translation of ribosome-related genes as reported previously (Akagi et al. 2023).

**Figure 2:**
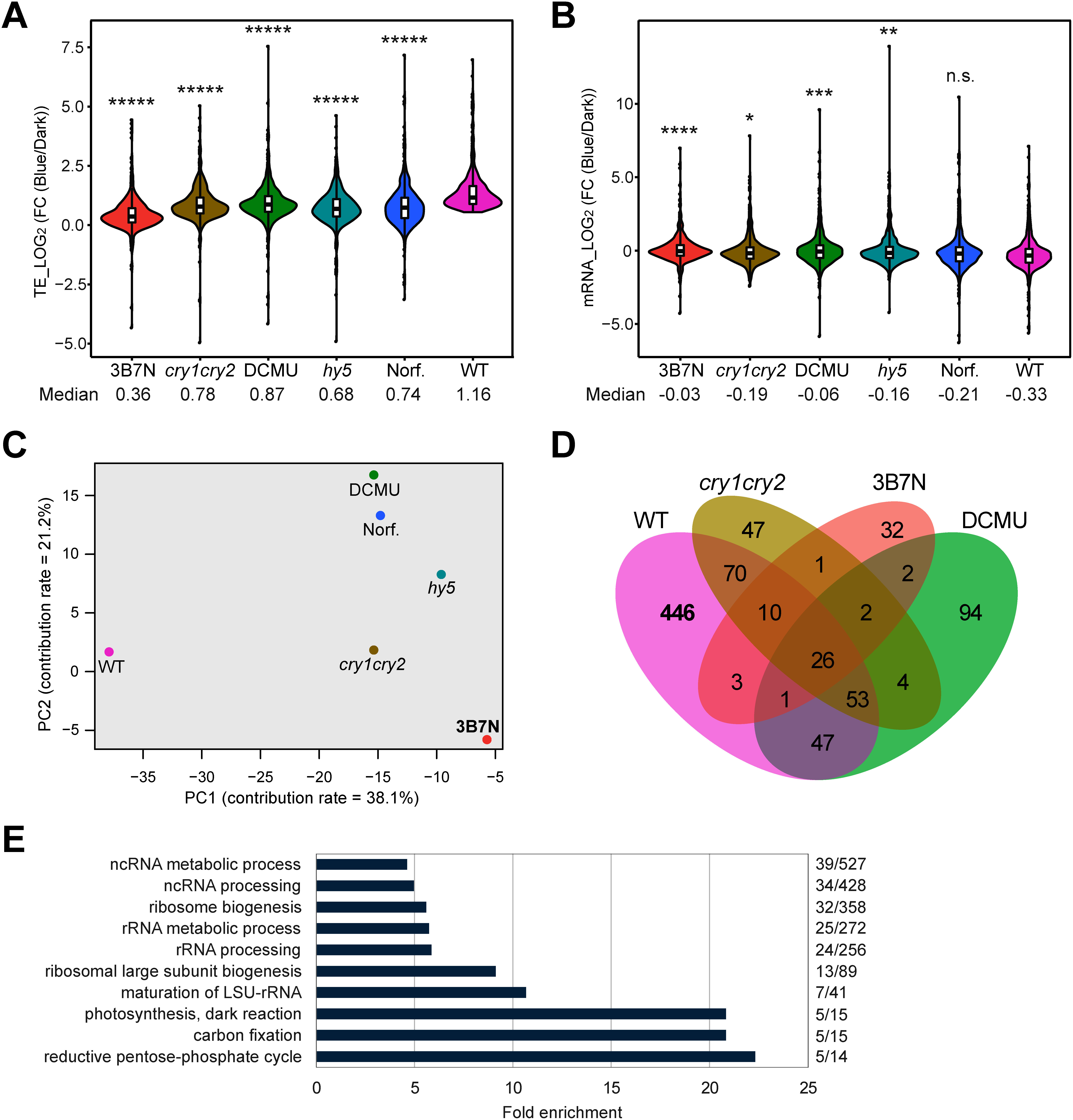
Figure 2. Effect of 3B7N treatment on gene expression of the 656 TE-up-regulated genes upon blue light exposure in the WT. (A) and (B), Changes of TE (A) and mRNA accumulation (B) of the 656 TE-up-regulated genes upon blue light exposure in the WT in WT, *hy5*, *cry1cry2* and 3B7N-, DCMU- and norflurazon (Norf.)-treated WT seedlings. Significant differences between the WT and each genotype and treatment in (A) and (B) were detected by Tukey’s HSD test (*p*-values *<10^−2^, **<10^−4^, ***<10^−5^, ****<10^−6^, *****<10^−7^, n.s.=not significant). (C) Principal component analysis for TE changes of the 656 genes. Contribution rates for PC1 and PC2 are 38.1% and 21.2%, respectively. (D) Overlap of TE-up-regulated genes between the WT, *cry1cry2*, DCMU and 3B7N. (E) GO analysis of the 446 genes upregulated upon blue light exposure in the WT but not in *cry1cry2*, DCMU or 3B7N. Numbers of enriched genes relative to total numbers of genes in each GO term are shown on the right.

It is surprising that 3B7N treatment and the *cry1cry2* and *hy5* mutations, which inhibit blue light signaling, and DCMU and norflurazon treatments, which inhibit photosynthesis, were closely plotted along the PC1 axis in the PCA analysis (Figure 2C), and that inhibition of either blue light signaling or photosynthesis negatively affected the translation of TE-up-regulated genes in the WT (Figure 2A). Inhibition of blue light signaling inhibits the induction of expression of nuclear genes for chloroplasts, resulting in a delay of early chloroplast formation, and reduced energy supply through photosynthesis (no carbon source in the medium) is a possible factor for reduced translation under inhibition of blue light signaling. However, it is unlikely that the energy supply from chloroplasts would significantly affect translation control in seedlings receiving only 3 h of light, where chloroplasts are not yet fully developed. It is expected that the crosstalk between blue light signaling, chloroplast formation and translation regulation will be clarified in the future.

It may be possible to regulate plant characteristics such as the blue light signaling pathway in a controlled manner through chemical treatment, which may lead to its use in crop applications. To exogenously inhibit CRY and control growth in response to light environments, 3B7N could be applied to crops that are recalcitrant to genetic modification. For example, 3B7N may be used to artificially induce shade-avoidance syndrome, which occurs under weak blue light intensity, induces some biological reactions such as stem elongation, accelerated flowering and suppressed leaf development, and finally affects crop productivity (Wang et al. 2020). To this end, it is necessary to continue to evaluate the potential of 3B7N through further analysis of its action and effects.

## Abbreviations

3B7N: 3-bromo-7-nitroindazole
CRY1: Cryptochrome 1
CRY2: Cryptochrome 2
DCMU: 3-(3,4-dichlorophenyl)-1,1-dimethylurea
GO: gene ontology
HY5: ELONGATED HYPOCOTYL5
ORF: open reading frames
WT: wild type

## Description of Supplementary Files

Supplementary Table S1. Numbers of raw and mapped reads of RNA-seq and Ribo-seq.

Supplementary Table S2. Numbers of TE- and mRNA-upregulated genes upon blue light exposure.

Supplementary Table S3. TE-upregulated genes upon blue light exposure in wild type seedlings.

Supplementary Table S4. mRNA-upregulated genes upon blue light exposure in wild type seedlings.

Supplementary Table S5. TE-upregulated genes upon blue light exposure in each genotype and treatment.

Supplementary Figure S1. Hierarchical clustering analysis of all biological replicates of RNA-seq and Ribo-seq.

Supplementary Figure S2. Correlation of expression changes upon blue light exposure between RiboTaper-predicted and Araport11-annotated ORFs.

Supplementary Figure S3. Validation of Ribo-seq analysis for 3B7N-treated seedlings.

Supplementary Figure S4. Heatmap for fold changes of TE and mRNA accumulation of the 656 TE-up-regulated genes upon blue light exposure in the WT.

## References

[RAkagi2023] Akagi C, Kurihara Y, Makita Y, Kawauchi M, Tsuge T, Aoyama T, Matsui M (2023) Translational activation of ribosome-related genes at initial photoreception is dependent on signals derived from both the nucleus and the chloroplasts in *Arabidopsis thaliana.* *J Plant Res* 136: 227–238 36658292 10.1007/s10265-022-01430-8

[RAnders2010] Anders S, Huber W (2010) Differential expression analysis for sequence count data. *Genome Biol* 11: R106 20979621 10.1186/gb-2010-11-10-r106 PMC3218662

[RCalviello2016] Calviello L, Mukherjee N, Wyler E, Zauber H, Hirsekorn A, Selbach M, Landthaler M, Obermayer B, Ohler U (2016) Detecting actively translated open reading frames in ribosome profiling data. *Nat Methods* 13: 165–170 26657557 10.1038/nmeth.3688

[RDobin2013] Dobin A, Davis CA, Schlesinger F, Drenkow J, Zaleski C, Jha S, Batut P, Chaisson M, Gingeras TR (2013) STAR: Ultrafast universal RNA-seq aligner. *Bioinformatics* 29: 15–21 23104886 10.1093/bioinformatics/bts635 PMC3530905

[RFujita2019] Fujita T, Kurihara Y, Iwasaki S (2019) The plant translatome surveyed by ribosome profiling. *Plant Cell Physiol* 60: 1917–1926 31004488 10.1093/pcp/pcz059

[RGangappa2016] Gangappa SN, Botto JF (2016) The multifaceted roles of HY5 in plant growth and development. *Mol Plant* 9: 1353–1365 27435853 10.1016/j.molp.2016.07.002

[RIngolia2009] Ingolia NT, Ghaemmaghami S, Newman JRS, Weissman JS (2009) Genome-wide analysis in vivo of translation with nucleotide resolution using ribosome profiling. *Science* 324: 218–223 19213877 10.1126/science.1168978 PMC2746483

[RKurihara2018] Kurihara Y, Makita Y, Kawashima M, Fujita T, Iwasaki S, Matsui M (2018) Transcripts from downstream alternative transcription start sites evade uORF-mediated inhibition of gene expression in. *Proc Natl Acad Sci USA* 115: 7831–7836 29915080 10.1073/pnas.1804971115 PMC6064979

[RKurihara2022] Kurihara Y, Makita Y, Kawauchi M, Kageyama A, Kuriyama T, Matsui M (2022) Intergenic splicing-stimulated transcriptional readthrough is suppressed by nonsense-mediated mRNA decay in Arabidopsis. *Commun Biol* 5: 1390 36539571 10.1038/s42003-022-04348-y PMC9768141

[RKurihara2020a] Kurihara Y, Makita Y, Shimohira H, Fujita T, Iwasaki S, Matsui M (2020a) Translational landscape of protein-coding and non-protein-coding RNAs upon light exposure in Arabidopsis. *Plant Cell Physiol* 61: 536–545 31794029 10.1093/pcp/pcz219

[RKurihara2020b] Kurihara Y, Makita Y, Shimohira H, Matsui M (2020b) Time-course transcriptome study reveals mode of bZIP transcription factors on light exposure in Arabidopsis. *Int J Mol Sci* 21: 1993 32183354 10.3390/ijms21061993 PMC7139404

[ROng2017] Ong WD, Okubo-Kurihara E, Kurihara Y, Shimada S, Makita Y, Kawashima M, Honda K, Kondoh Y, Watanabe N, Osada H, et al. (2017) Chemical-induced inhibition of blue light-mediated seedling development caused by disruption of upstream signal transduction involving cryptochromes in *Arabidopsis thaliana.* *Plant Cell Physiol* 58: 95–105 28011868 10.1093/pcp/pcw181

[ROrth2017] Orth C, Niemann N, Hennig L, Essen LO, Batschauer A (2017) Hyperactivity of the Arabidopsis cryptochrome (cry1) L407F mutant is caused by a structural alteration close to the cry1 ATP-binding site. *J Biol Chem* 292: 12906–12920 28634231 10.1074/jbc.M117.788869 PMC5546031

[RPark2017] Park JH, Tran LH, Jung S (2017) Perturbations in the photosynthetic pigment status result in photooxidation-induced crosstalk between carotenoid and porphyrin biosynthetic pathways. *Front Plant Sci* 8: 1992 29209351 10.3389/fpls.2017.01992 PMC5701815

[RPetrillo2014] Petrillo E, Godoy Herz MA, Fuchs A, Reifer D, Fuller J, Yanovsky MJ, Simpson C, Brown JW, Barta A, Kalyna M, et al. (2014) A chloroplast retrograde signal regulates nuclear alternative splicing. *Science* 344: 427–430 24763593 10.1126/science.1250322 PMC4382720

[RTrapnell2009] Trapnell C, Pachter L, Salzberg SL (2009) TopHat: Discovering splice junctions with RNA-Seq. *Bioinformatics* 25: 1105–1111 19289445 10.1093/bioinformatics/btp120 PMC2672628

[RWang2020] Wang X, Gao X, Liu Y, Fan S, Ma Q (2020) Progress of research on the regulatory pathway of the plant shade-avoidance syndrome. *Front Plant Sci* 11: 439 32351535 10.3389/fpls.2020.00439 PMC7174782

[RXiao2022] Xiao Y, Chu L, Zhang Y, Bian Y, Xiao J, Xu D (2022) HY5: A pivotal regulator of light-dependent development in higher plants. *Front Plant Sci* 12: 800989 35111179 10.3389/fpls.2021.800989 PMC8801436

