# Supplementary material for: The blue light signaling inhibitor 3-bromo-7-nitroindazole affects gene translation at the initial reception of blue light in young Arabidopsis seedlings
Source: Plant Biotechnol (Tokyo). 2024 Jun 25;41(2):153–7. doi: 10.5511/plantbiotechnology.24.0323a (PMC11500569; doi:10.5511/plantbiotechnology.24.0323a)
Supplement: Supplementary Data [file plantbiotechnology-41-2-24.0323a-s001.pdf]

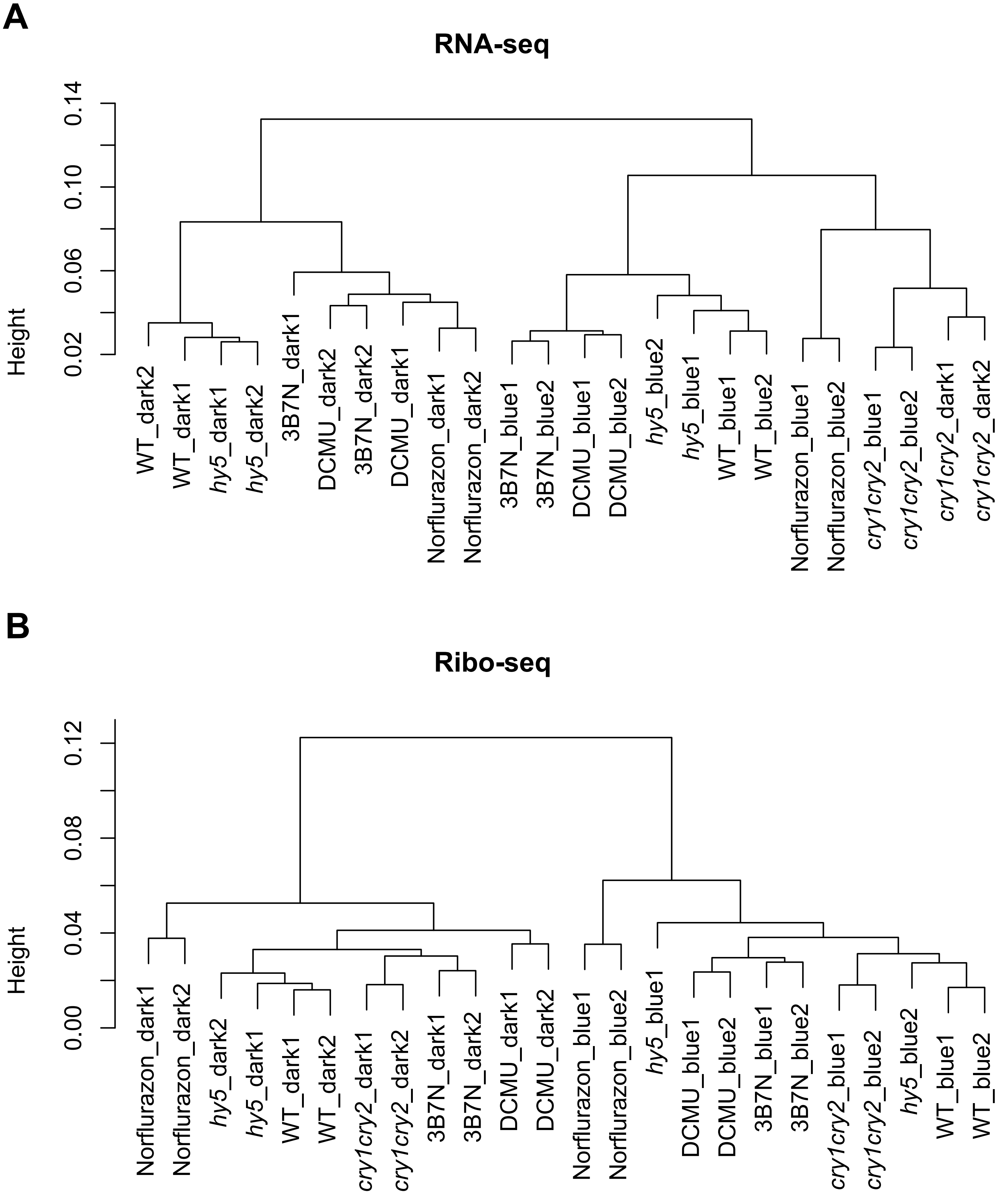

Supplementary Figure S1. Hierarchical clustering analysis of all biological replicates of RNA-seq (A) and Ribo-seq (B). Normalized read counts of 31,209 mRNA-generating genes were used for the depiction. Similarity of normalized read count between samples was defined by the Spearman rank correlation coefficient. The cluster dendrogram was depicted using Ward's method.

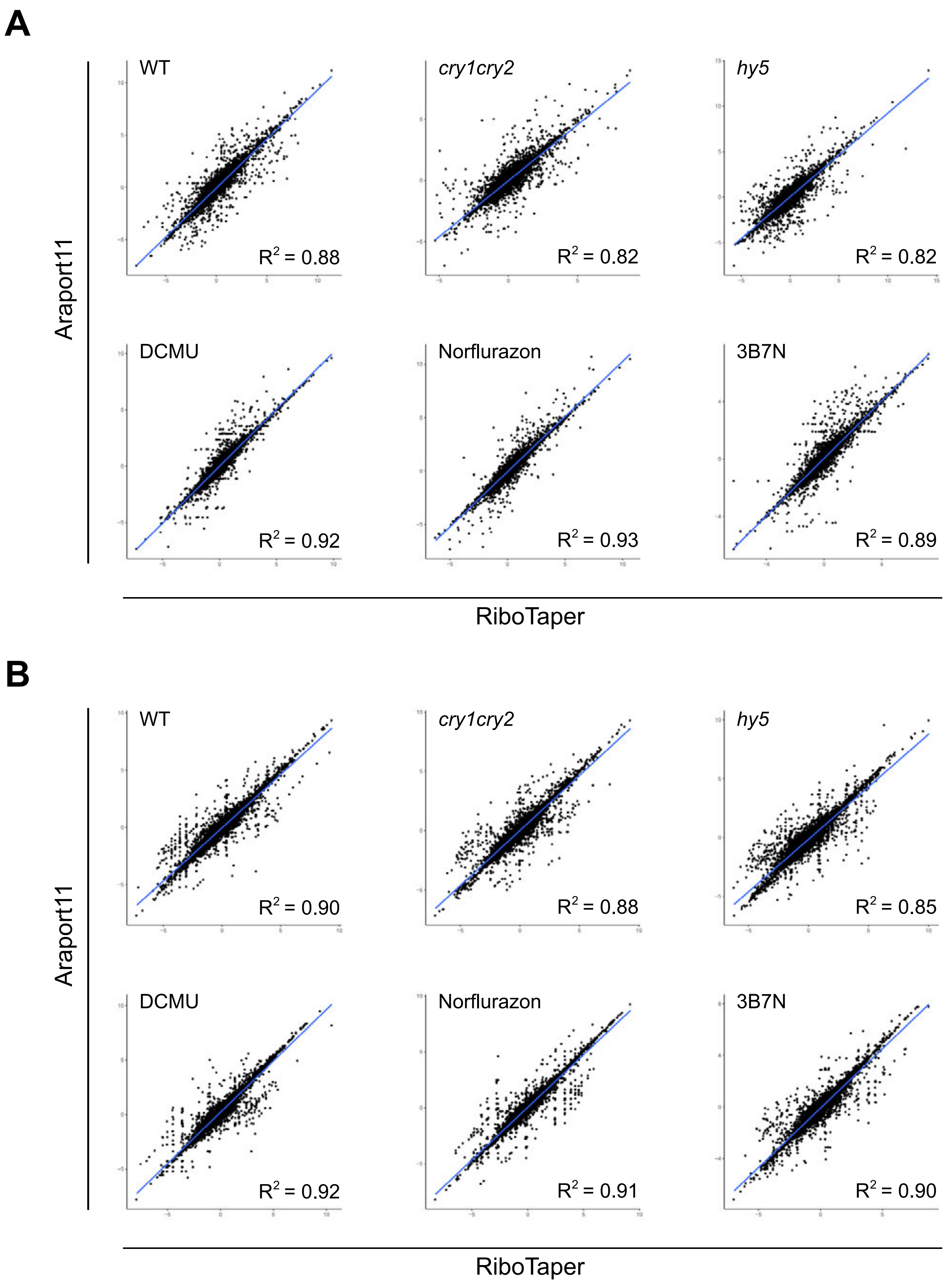

Supplementary Figure S2. Correlation of expression changes upon blue light exposure between RiboTaper-predicted and Araport11-annotated ORFs. (A) RNA-seq, (B) Ribo-seq.

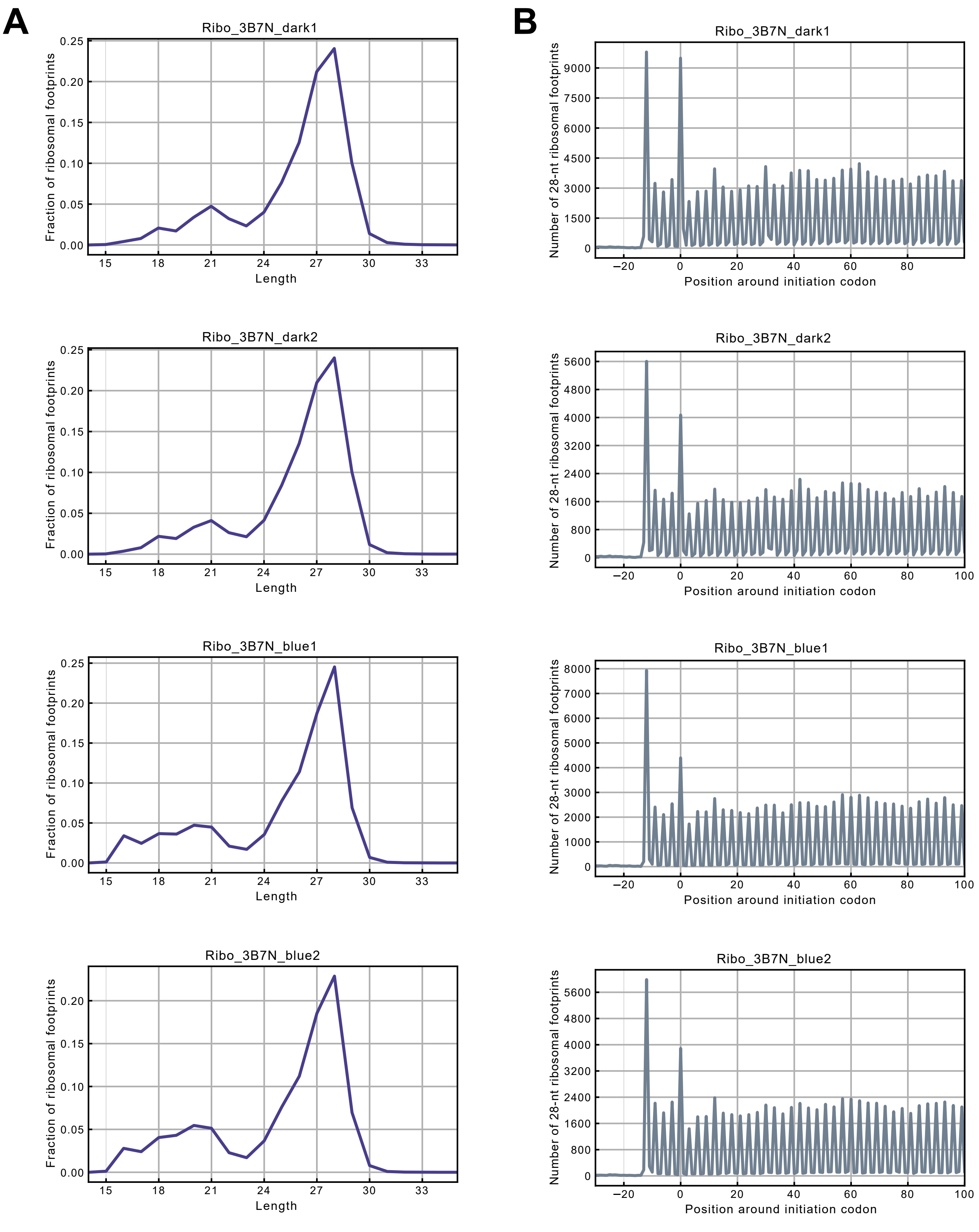

Supplementary Figure S3. Validation of Ribo-seq analysis for 3B7N-treated seedlings. (A) Length distribution of ribosomal footprints in 3B7N-treated seedlings. (B) Metagene analysis for 3-nucleotide periodicity of ribosomal footprints on the ORFs in 3B7N-treated seedlings.

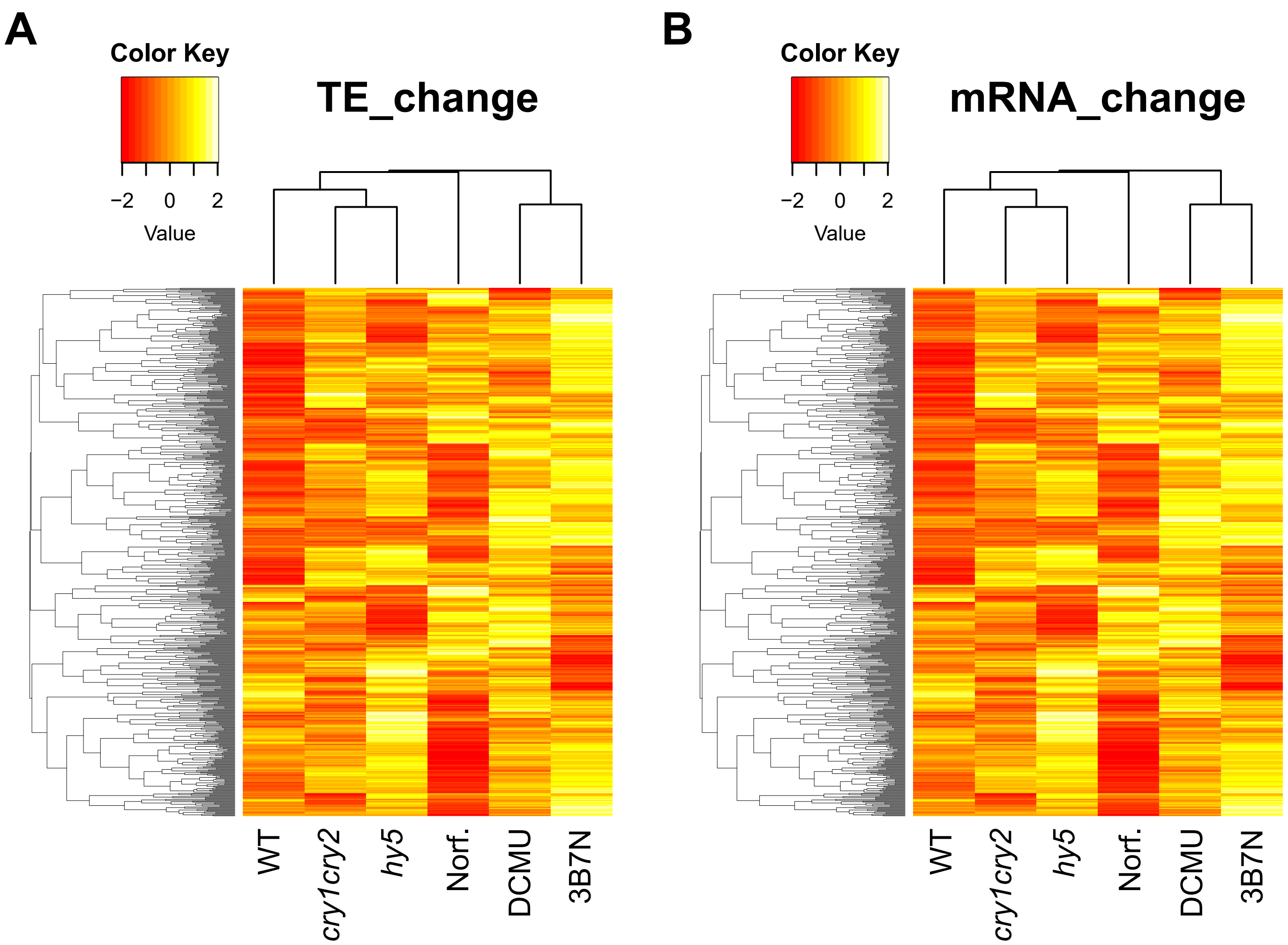

Supplementary Figure S4. (A) Heatmap for TE fold changes of the 656 genes for which TEs were upregulated upon blue light exposure in the WT. (B) Heatmap for mRNA accumulation fold changes of the 656 genes. The means of the two replicates were used for each plot.
